# 双荧光标记的人高骨转移肺腺癌细胞株的建立及其转录组学特征分析

**DOI:** 10.3779/j.issn.1009-3419.2024.101.09

**Published:** 2024-04-20

**Authors:** Yue LU, Rong QIU, Yan DENG, Xingyu LIU, Yuzhen DU

**Affiliations:** ^1^201306 上海，上海海洋大学水产与生命学院; ^1^College of Fisheries and Life Science, Shanghai Ocean University, Shanghai 201306, China; ^2^201306 上海，上海交通大学医学院附属第六人民医院医学检验科; ^2^Department of Laboratory Medicine, Shanghai Jiao Tong University Affiliated Sixth People’s Hospital, Shanghai 201306, China

**Keywords:** 肺肿瘤, 骨转移, 动物模型, 细胞株, 转录组学分析, Lung neoplasms, Bone metastasis, Animal models, Cell line, Transcriptomic analysis

## Abstract

**背景与目的:**

骨是肺腺癌常见的转移部位，但肺腺癌骨转移的机制尚不明确。目前肺腺癌骨转移机制研究缺乏易于示踪且稳定高骨转移的肺腺癌细胞模型，因此，本研究旨在建立绿色荧光蛋白（green fluorescent protein, GFP）和萤火虫荧光素酶（firefly luciferase, LUC）双标记的人高骨转移肺腺癌细胞株，为肺腺癌骨转移的研究提供新的实验工具。

**方法:**

人肺腺癌细胞系A549-GFP-LUC经左心室注射至裸鼠体内构建骨转移模型，经连续3次体内驯化，获取人高骨转移肺腺癌细胞株A549-GFP-LUC-BM3；CCK-8（cell counting kit-8）、克隆形成实验比较A549-GFP-LUC-BM3细胞株和亲本细胞的体外增殖能力，划痕实验、Transwell实验以及Western blot比较迁移和侵袭能力；并进一步将A549-GFP-LUC-BM3细胞和亲本细胞行测序转录组学分析。

**结果:**

成功建立人高骨转移肺腺癌细胞A549-GFP-LUC-BM3，相较于亲本细胞，该细胞骨转移发生率显著提高，且体外增殖、迁移和侵袭能力显著增强。转录组学测序结果显示，相较于亲本细胞，A549-GFP-LUC-BM3细胞中共筛选到差异基因2954个，其中1021个基因上调，1933个基因下调；基因本体（Gene Ontology, GO）功能富集显示差异基因主要定位于细胞外周、质膜以及细胞外基质等细胞组分，分子功能主要富集在信号受体结合、钙离子结合和细胞外基质结构成分等，生物过程富集在细胞黏附和生物黏附等；京都基因与基因组百科全书（Kyoto Encyclopedia of Genes and Genomes, KEGG）富集分析显示差异基因在细胞色素P450（cytochrome P450, CYP）对外源性物质的代谢、视黄醇代谢、细胞黏附分子、CYP对药物代谢、类固醇激素的生物合成以及核因子κB（nuclear factor kappa B, NF-κB）信号通路上显著富集。

**结论:**

成功建立GFP和LUC双标记的人高骨转移肺腺癌细胞株，该细胞株在生物学行为水平和转录组测序水平均提示具有高骨转移潜能。

## Abstract (en)

**Background and objective:**

Bone is a common site for metastasis in lung adenocarcinoma, but the mechanism behind lung adenocarcinoma bone metastasis is still unclear. And currently, there is a lack of easily traceable and stable lung adenocarcinoma bone metastasis cell models, which limits the research on the mechanism of lung adenocarcinoma bone metastasis. The establishment of human lung adenocarcinoma cell line that are highly metastatic to bone, labeled with green fluorescent proteins (GFP) and fireflies luciferase (LUC), along with transcriptomic characterization, would be beneficial for research on lung adenocarcinoma bone metastasis and provide new experimental methods.

**Methods:**

The human lung adenocarcinoma cell line A549-GFP-LUC was injected into nude mice via the left ventricle to construct a bone metastasis model, and was domesticated in vivo for three consecutive times to obtain the human high bone metastasis lung adenocarcinoma cell line A549-GFP-LUC-BM3; cell counting kit-8 (CCK-8), colony formation assay, scratch wound assays, Transwell assay and Western blot were used to compare the proliferation and invasion abilities of A549-GFP-LUC-BM3 with the parental cells. A549-GFP-LUC-BM3 cells and parental cells were further analyzed by transcriptomic sequencing.

**Results:**

Human high-bone metastatic lung adenocarcinoma cells A549-GFP-LUC-BM3 was successfully established. Compared to parental cells, this cells exhibited a significantly higher incidence of bone metastasis and enhanced in vitro proliferation, migration, and invasion abilities. Transcriptomic sequencing results revealed that the A549-GFP-LUC-BM3 cell line had 2954 differentially expressed genes compared to the parental cells, with 1021 genes up-regulated and 1933 genes down-regulated. Gene Ontology (GO) functional enrichment analysis indicated that the differentially expressed genes were primarily localized in cellular components such as the cell periphery. The molecular functions identified as significantly enriched included signaling receptor activity, calcium ion binding, and extracellular matrix structural constituent. Additionally, the biological processes found to be enriched were cell adhesion and biological adhesion. The enrichment analysis conducted using the Kyoto Encyclopedia of Genes and Genomes (KEGG) revealed that the differentially expressed genes were primarily involved in the metabolism of xenobiotics by cytochrome P450, retinol metabolism, drug metabolism-cytochrome P450, cell adhesion molecules, steroid hormone biosynthesis, and the nuclear factor kappa B (NF-κB) signaling pathway.

**Conclusion:**

The highly bone-metastatic human lung adenocarcinoma cell line with GFP and luciferase double labeling was successfully established. The biological behavior and transcriptome sequencing of the cell line suggest that it has a high bone-metastatic potential.

肺腺癌是肺癌常见的病理亚型^[1]^，约占肺癌的40%^[2]^。远端转移是肿瘤预后差的关键，肺腺癌常转移至骨^[3]^。但是肺腺癌骨转移的具体机制仍不清楚。骨转移动物模型是研究肿瘤骨转移机制的重要工具，但现有肺腺癌骨转移细胞株造模效率不高，且不能快速、有效地在动物体内观察到肿瘤细胞转移的动态过程，因此建立双荧光标记的高骨转移肺腺癌细胞株可以为肺腺癌骨转移的研究提供新的实验手段。

本研究拟用绿色荧光蛋白（green fluorescent protein, GFP）和萤火虫荧光素酶（firefly luciferase, LUC）双标记的人肺腺癌A549细胞株（A549-GFP-LUC）通过裸鼠左心室注射构建肺腺癌骨转移模型，经体内驯化3次后，建立GFP和LUC双标记的人高骨转移肺腺癌细胞株，并在细胞生物学行为水平和转录组测序水平分析其高骨转移特性。

## 1 材料与方法

### 1.1 细胞系和主要试剂

人肺腺癌细胞系A549-GFP-LUC购自上海中乔新舟生物科技有限公司；BALB/c裸鼠（雌性，18-22 g）购自北京斯贝福生物技术有限公司；DMEM/F-12（1:1）培养基、青霉素-链霉素溶液（P/S）、0.25% Trypsin-EDTA购自美国Gibco公司；胎牛血清（FBS）购自上海ExCell Bio公司；Matrigel基质胶、8 μm孔径的Transwell小室购自美国Corning公司；BCA蛋白浓度测定试剂盒（增强型）、RIPA裂解液、结晶紫染色液、CCK-8（cell counting kit-8）试剂盒购自上海Beyotime公司；ECL超敏发光检测试剂购自上海Epizyme公司；E-cadherin（No.A20798, 1:1000）、N-cadherin（No.A19083, 1:1000）、Vimentin（No.A19607, 1:1000）、β-actin（No.AC026, 1:1000）抗体和HRP Goat Anti-Rabbit IgG （H+L）（No.AS014, 1:10,000）抗体均购自武汉ABclonal公司；I型胶原酶购自美国Sigma公司。

### 1.2 细胞培养

人肺腺癌细胞株A549-GFP-LUC和人高骨转移肺腺癌细胞株A549-GFP-LUC-BM3用含10% FBS的DMEM/F-12（1:1）培养基在37 ^o^C、5% CO_2_培养箱中恒温培养。

### 1.3 双荧光标记的人高骨转移肺腺癌细胞株A549-GFP-LUC-BM3的建立

借鉴文献^[4]^报道高转移细胞株的构建，通过左心室注射人肺腺癌A549-GFP-LUC细胞，经体内驯化3次后，建立双荧光标记的人高骨转移肺腺癌细胞株，命名为A549-GFP-LUC-BM3，该细胞株已申请专利（申请号为202311464185.7）。

BALB/c裸鼠适应性饲养1周后进行造模，每次实验随机选取6只裸鼠。取对数生长期的A549-GFP-LUC细胞消化，PBS重悬并调整细胞浓度为1×10^7^个/mL；用异弗烷麻醉裸鼠，吸取100 μL细胞重悬液注射至裸鼠左心室，在SPF级条件下饲养35 d，小动物活体成像观察转移情况。根据活体成像结果，取发生转移的四肢长骨用眼科剪剪碎，加入2 mg/mL I型胶原酶，在37 ^o^C水浴锅中消化3次，每次25 min，收集消化后的上清800 rpm离心5 min，用含20% FBS和2% PS的培养基重悬细胞沉淀进行培养，3-4 d更换一次新鲜培养基，待细胞状态稳定后换成含10% FBS的培养基传代培养（命名为A549-GFP-LUC-BM1），完全贴壁长满后，消化重悬，通过流式荧光分选技术（fluorescence-activated cell sorting, FACS）分选GFP阳性的细胞，再次裸鼠左心室注射，循环3次获得A549-GFP-LUC-BM3细胞（图1A）。本研究中动物实验已通过上海交通大学医学院附属第六人民医院动物伦理委员会的审批，审批号为2023-YS-093。

**图 1:**
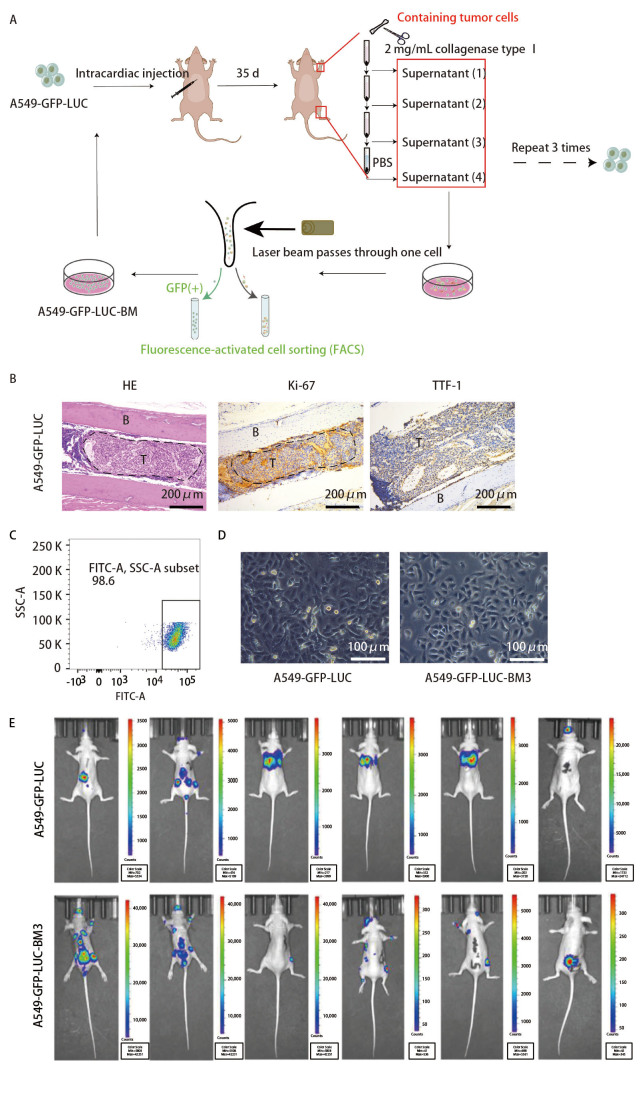
双荧光标记的人高骨转移肺腺癌细胞株A549-GFP-LUC-BM3的建立。A：双荧光标记的人高骨转移细胞构建流程图；B：采用FACS分选A549-GFP-LUC-BM3细胞；C：A549-GFP-LUC和A549-GFP-LUC-BM3细胞形态观察；D、E：A549-GFP-LUC和A549-GFP-LUC-BM3细胞体内骨转移发生率。

### 1.4 苏木素-伊红（hematoxylin-eosin, HE）和免疫组化染色

裸鼠脱颈处死，取胫骨固定在4%多聚甲醛24 h以上，EDTA脱钙2周，梯度酒精脱水、石蜡包埋后切片4 μm，HE、Ki-67及TTF-1染色，倒置显微镜下拍照（×100）。

### 1.5 A549-GFP-LUC-BM3的细胞生物学特性

取对数生长期的A549-GFP-LUC-BM3细胞和亲本A549-GFP-LUC细胞消化重悬，每孔2×10^5^个细胞接种于6孔板，待细胞密度达到70%时，在倒置显微镜下拍摄细胞形态。以A549-GFP-LUC亲本细胞为对照组，进行增殖、迁移和侵袭能力实验。

#### 1.5.1 CCK-8法检测细胞增殖能力

取对数生长期的A549-GFP-LUC-BM3细胞和亲本细胞消化重悬，每孔3000个细胞接种于96孔板，每组细胞5个复孔，分别在贴壁12、24、48、72 h后加入CCK-8试剂，在培养箱中放置1 h，测定450 nm下的光密度值。

#### 1.5.2 克隆形成实验

每孔800个细胞接种于6孔板，3 d更换一次完全培养基，培养14 d，4%多聚甲醛固定30 min，结晶紫染色7 min，PBS洗涤3次，晾干拍照；使用Image J软件对细胞克隆形成的集落进行计数。

#### 1.5.3 划痕实验

每孔3×10^5^个细胞接种于6孔板，用灭菌的10 μL枪头垂直划痕，弃培养基后PBS洗涤3次，洗净孔板中的漂浮细胞，每孔加入2 mL基础培养基，分别于0和24 h时在显微镜下拍照，使用Image J软件进行统计。

#### 1.5.4 Transwell实验

侵袭实验：将Matrigel基质胶和DMEM/F-12（1:1）培养基以1:4的比例稀释，取50 μL加入小室中，放置培养箱中30 min，待基质胶凝固，再取对数生长期的细胞消化，加入基础培养基重悬，计数5×10^4^个细胞接种于小室内，24 h后4%多聚甲醛室温固定30 min，结晶紫染色7 min，PBS清洗小室，在倒置显微镜下随机拍摄3个视野，使用Image J软件对侵袭的细胞进行计数。迁移实验：小室内不加Matrigel基质胶，细胞加入小室12 h后终止实验，其余实验条件和操作同上。

#### 1.5.5 Western blot

提取A549-GFP-LUC-BM3细胞和亲本细胞总蛋白，BCA法测定蛋白浓度，两组各取20 μg总蛋白配制上样体系，在10% SDS-PAGE凝胶电泳中分离，恒流350 mA转PVDF膜，5%脱脂牛奶室温封闭2 h，一抗4 ^o^C过夜，TBST洗涤3次，二抗室温孵育1 h，使用超敏ECL显影，使用Image J软件分析蛋白条带的灰度值。

### 1.6 转录组测序及生物信息分析

#### 1.6.1 转录组测序

使用Trizol提取A549-GFP-LUC细胞和A549-GFP-LUC-BM3细胞总RNA，每个细胞3份样本，提取过程中保证RNA质量，对RNA质检合格后，用带有Oligo（dT）的磁珠富集mRNA，加入裂解缓冲液随机打断mRNA；然后用片段化后的mRNA作为模板合成cDNA第一链；加入适量缓冲液、dNTPs和DNA聚合酶I合成cDNA第二链；使用磁珠纯化双链cDNA，再进行末端修复、加A尾以及连接测序接头，之后用磁珠进行片段筛选，聚合酶链式反应（polymerase chain reaction, PCR）扩增，得到最终的cDNA文库。检测文库质量，库检合格后，在测序平台（华大智造DNBSEQ-T7）测序。

#### 1.6.2 差异基因获取及富集分析

采用DESeq2方法对样品进行差异基因分析，利用R包在|log2(foldchange)|>1，P<0.05的条件下，筛选差异基因。根据差异基因检测结果进行下一步富集分析。

差异基因在DAVID数据库（https://david.ncifcrf.gov/）进行基因本体（Gene Ontology, GO）富集分析和京都基因与基因组百科全书（Kyoto Encyclopedia of Genes and Genomes, KEGG）通路富集分析。

### 1.7 统计学分析

采用GraphPad 9.0.6软件对实验结果进行统计学分析及作图，每个实验重复3次。两组间比较采用t检验，P<0.05为差异有统计学意义。

## 2 结果

### 2.1 双荧光标记的人高骨转移肺腺癌细胞株A549-GFP-LUC-BM3的建立

通过裸鼠左心室注射A549-GFP-LUC细胞构建肺腺癌骨转移模型（图1A），病理结果显示，肿瘤细胞成功转移至胫骨（图1B），分离提取裸鼠四肢长骨肿瘤转移灶中的肿瘤细胞扩大培养，FACS分选出GFP阳性细胞（图1C），经3次体内外循环后，成功建立高骨转移肺腺癌细胞，命名A549-GFP-LUC-BM3，短串联重复序列（short tandem repeat, STR）鉴定其分型为A549。显微镜下观察A549-GFP-LUC-BM3细胞形态，呈多边形，上皮样（图1D），与亲本细胞相比细胞形态无明显变化。生物发光成像结果显示（图1E），注射A549-GFP-LUC亲本细胞，6只裸鼠中有1只发生骨转移，骨转移发生率为16.7%，在体内3次驯化过程中，骨转移发生率逐渐提高（表1），注射A549-GFP-LUC-BM3细胞的6只裸鼠全部发生骨转移，并且在股骨、胫骨及腰椎等位置发生多发骨转移，骨转移率达到100.0%，以上结果表明成功建立高骨转移肺腺癌细胞株。

**表 1:** 裸鼠造模骨转移发生率比较

Cell	Number of experimental nude mice	Number of nude mice with bone metastases	The number of bone metastasis sites	Incidence of bone metastases
A549-GFP-LUC	6	1	3	16.7%
A549-GFP-LUC-BM1	6	2	5	33.3%
A549-GFP-LUC-BM2	6	4	13	66.7%
A549-GFP-LUC-BM3	6	6	15	100.0%

The occurrence of spinal and limb long bone metastasis in nude mice were statistically analyzed.

### 2.2 亲本细胞与A549-GFP-LUC-BM3细胞的生物学特性比较

#### 2.2.1 A549-GFP-LUC-BM3细胞增殖能力增强

检测A549-GFP-LUC-BM3细胞增殖能力的变化，CCK-8实验结果显示（图2A），相较于亲本细胞，A549-GFP-LUC-BM3细胞增殖能力显著提高。克隆形成实验的结果表明（图2B），A549-GFP-LUC-BM3细胞的克隆形成数明显多于亲本细胞，说明高骨转移肺腺癌细胞株具有更强的增殖能力。

**图 2:**
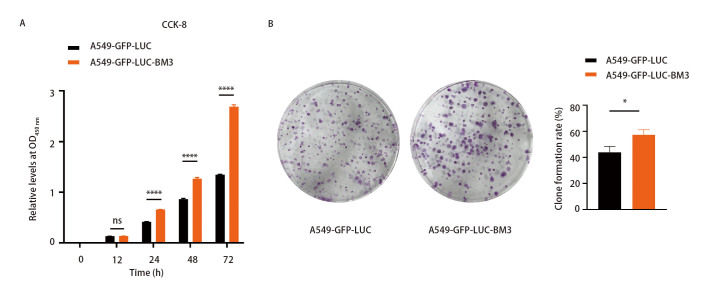
A549-GFP-LUC-BM3细胞增殖能力增强。A：CCK-8检测A549-GFP-LUC和A549-GFP-LUC-BM3细胞的增殖能力；B：克隆形成实验检测A549-GFP-LUC和A549-GFP-LUC-BM3细胞克隆形成能力。^* ^P<0.05；^****^P<0.0001。

#### 2.2.2 A549-GFP-LUC-BM3细胞迁移和侵袭能力增强

采用细胞划痕实验对比高骨转移肺腺癌细胞株和亲本细胞的横向迁移能力，结果如图3A所示，相较于亲本细胞，A549-GFP-LUC-BM3细胞在无血清的培养基中划痕愈合速度更快。通过Transwell实验检测A549-GFP-LUC-BM3细胞迁移和侵袭能力的变化，结果如图3B所示，A549-GFP-LUC-BM3细胞迁移和侵袭能力显著强于亲本细胞。

**图 3:**
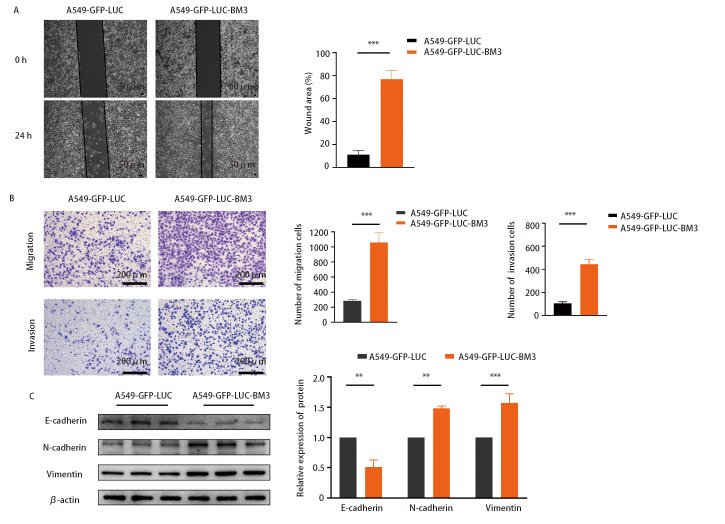
A549-GFP-LUC-BM3细胞迁移和侵袭能力增强。A：划痕实验表明A549-GFP-LUC-BM3细胞的横向迁移能力强于亲本A549-GFP-LUC细胞。右侧为横向迁移面积统计图；B：Transwell实验表明A549-GFP-LUC-BM3细胞迁移和侵袭能力强于亲本A549-GFP-LUC细胞。右侧为迁移和侵袭后细胞数目统计图；C：采用Western blot检测EMT标志蛋白E-cadherin、N-cadherin、Vimentin的表达情况，右侧为蛋白相对表达量统计图。^**^P<0.01；^***^P<0.001。

肿瘤细胞经上皮间充质转化（epithelial-mesenchymal transition, EMT）迁移、侵袭能力增强^[5]^，因此检测A549-GFP-LUC-BM3细胞和A549-GFP-LUC细胞EMT标志蛋白E-cadherin、N-cadherin、Vimentin的表达，结果如图3C所示，相比于亲本细胞，A549-GFP-LUC-BM3细胞中N-cadherin、Vimentin的表达显著提高，E-cadherin表达显著下降，提示A549-GFP-LUC-BM3细胞发生EMT、迁移和侵袭能力增强。

### 2.3 亲本细胞与A549-GFP-LUC-BM3细胞的转录组学测序分析

#### 2.3.1 差异基因筛选

为探究A549-GFP-LUC-BM3细胞相较于亲本细胞在基因表达水平的变化，进行转录组学测序分析。使用DESeq2工具，以P<0.05，|log2(foldchange)|>1作为筛选差异表达基因的标准，结果显示，相对于亲本细胞，A549-GFP-LUC-BM3细胞共筛选到2954个差异表达的基因，其中1021个基因表达上调，1933个基因表达下调（图4A，4B）。聚类分析结果显示两组重复样品的差异基因表达基本一致（图4C）。

**图 4:**
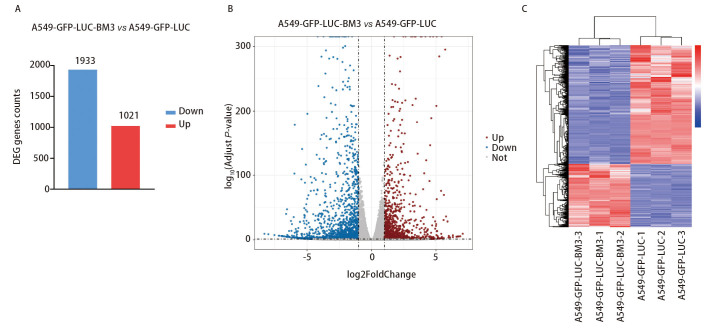
差异基因表达情况。A：差异基因柱状统计图；B：火山图；C：聚类热图。

#### 2.3.2 GO和KEGG富集分析

接下来对A549-GFP-LUC-BM3细胞和亲本细胞的差异表达基因进行GO富集分析，以了解A549-GFP-LUC-BM3细胞差异表达基因的功能。GO富集分析由细胞组分（cellular component, CC）、分子功能（molecular function, MF）和生物过程（biological process, BP）3个层次组成。结果显示，A549-GFP-LUC-BM3细胞差异表达基因在细胞组分主要定位于细胞外周、质膜、细胞外基质等组分上，分子功能主要富集在钙离子结合、信号受体结合和细胞外基质结构成分等方面，生物过程富集在细胞黏附和生物黏附等过程（图5A）。对两组细胞的差异表达基因进行KEGG pathway富集分析以进一步了解差异基因具体涉及哪些信号通路。结果显示，差异基因主要在细胞色素P450（cytochrome P450, CYP）对外源性物质的代谢、视黄醇代谢、CYP对药物代谢、细胞黏附分子、类固醇激素的生物合成以及核因子κB（nuclear factor kappa B, NF-κB）信号通路等通路显著富集（图5B）。

**图 5:**
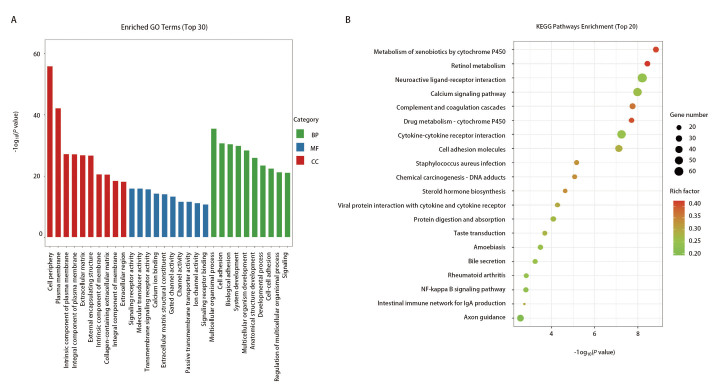
差异表达基因GO和KEGG通路富集分析。A：GO富集分析；B：KEGG pathway富集分析。

## 3 讨论

为获得高骨转移的肺腺癌细胞株，将人肺腺癌细胞A549-GFP-LUC注射至裸鼠左心室，分离骨转移灶的肿瘤细胞进行3次体内驯化，成功建立了GFP和LUC双标记的高骨转移A549-GFP-LUC-BM3细胞株。该细胞株在裸鼠体内可发生腰椎、胫骨及股骨等多发骨转移，骨转移发生率可达100.0%，易于在动物体内示踪。

连续体内驯化是建立器官特异性高转移潜能肿瘤细胞的有效方式^[6,7]^。常用于建模的肺腺癌细胞有A549、H1299和SPC-A-1等，其中A549细胞相比于其他肺腺癌细胞，具有较高的骨转移发生率^[8]^。目前肺腺癌骨转移动物模型的体内注射方式有胫骨注射、原位注射和左心室注射等^[9]^，胫骨注射易于建模，但并不符合肺腺癌发生骨转移的变化过程^[10]^；肺癌细胞原位注射方式虽然可以更好地模拟肺癌转移过程，但是肺癌细胞原位种植容易产生并发症，并且实验周期长，成模率较低且不稳定^[10]^；肺癌细胞左心室注射方式造模可以让细胞经左心室进入血液循环，经过黏附、迁移等过程，最终定植在靶器官，形成转移灶，从而模拟肿瘤的血道转移过程^[9]^，相比于其他注射方式，左心室注射造模更接近肺腺癌发生转移的临床特征。因此，本研究采用左心室注射方式，注射肿瘤细胞35 d后，观察到骨转移，选择易于分离的四肢长骨分离提取肿瘤细胞，经3次体内驯化，成功建立人高骨转移肺腺癌细胞株A549-GFP-LUC-BM3，该细胞株骨转移发生率达到100.0%，并且在生物学水平上证实该细胞株体外增殖和迁移、侵袭能力显著强于亲本细胞。以往报道的高骨转移肺腺癌细胞的骨转移观察方式局限于放射性核素^[11,12]^，缺乏简易的检测手段，本研究所构建GFP和LUC双标记的高骨转移肺腺癌细胞株易于在动物体内示踪，且便于在动物体内观察肿瘤细胞的动态转移过程，是良好的细胞实验模型。

为探究高骨转移肺腺癌细胞株基因表达谱的变化，本研究对高骨转移肺腺癌细胞株和亲本细胞进行转录组学测序分析，共筛选到2954个差异表达的基因，其中1021个基因表达上调，1933个基因表达下调，GO分析结果显示差异基因与肿瘤细胞转移性传播的特征有关，包括肿瘤细胞内在特征，如细胞黏附和生物黏附等，以及可以反映肿瘤细胞与肿瘤微环境之间相互作用的特征，包括信号受体结合和细胞外基质结构成分等，这些功能变化对转移以及肿瘤在微环境中的定植十分重要^[13]^。KEGG pathway富集分析中发现差异基因富集在CYP外源物质代谢、视黄醇代谢、CYP药物代谢、NF-κB信号通路以及类固醇激素的生物合成等通路。CYP酶广泛参与肿瘤的生长、进展和转移^[14]^，CYP家族的CYP2J2在乳腺癌细胞中过表达可以促进骨形态发生蛋白受体1B（bone morphogenetic protein receptor 1, BBMPR1B）的表达上调进而促进乳腺癌细胞迁移^[15]^；CYP1A1也是CYP家族中的一个重要的酶，在乳腺癌中，抑制CYP1A1/NF-κB轴，可以抑制乳腺癌细胞的转移^[16]^，本研究转录组测序分析发现CYP1A1在高骨转移肺腺癌细胞中表达上调，提示CYP1A1可能在肺腺癌骨转移的发生发展中发挥作用，还需进一步的实验验证。NF-κB信号通路参与肿瘤细胞进展，包括转移、干性和耐药等^[17]^，NF-κB抑制剂可显著降低非小细胞肺癌的EMT、迁移和侵袭能力^[18]^，环状RNA circIKBKB过度激活NF-κB信号通路促进乳腺癌骨转移^[19]^，以上提示NF-κB信号通路与骨转移相关。

综上所述，本研究成功建立GFP和LUC双标记的人高骨转移肺腺癌细胞株，该细胞株来源于A549细胞系，骨转移发生率可达100.0%，在生物学水平和转录水平均提示有高转移潜能；该细胞模型自带荧光标记，易于观察肿瘤在活体内的存在状态，为在体内的肺腺癌骨转移机制研究以及药物治疗效果研究等提供了方便，有望成为肺腺癌骨转移机制研究新的实验工具。
